# Domestic Summary

**Published:** 1838-01-17

**Authors:** 


### DOMESTIC SUMMARY.

At the annual election for officers of the Philadelphia Medical Society, held January 5th, 1838, Nathaniel Chapman, M. D. was elected President of the Society, in the room of Dr. Physick, deceased. Samuel Jackson, M. D., and Thomas Harris, M. D. were chosen Vice Presidents.

*Dr. Mott.*—We notice in the last No. of the *Boston Med. and Surg. Journal,* a letter from Dr. Mott, of New York,—now in Europe travelling for his health—in which he announces the projection and commencement of a practical treatise on Surgery. "It will be upon the basis of relative anatomy, as I have been in the habit of teaching for some years, and the immense value of which I am more and more convinced of the longer I live, and the more I see. Upon this I shall engraft my views of surgical pathology, and my experience. It will not be my object to load it with opinions of others, by liberal quotations, by which I might display my reading and my surgical erudition, and make it a work of reference for other men's opinions. It shall contain my own opinions, and a simple narration of my own experience." So excellent a plan, executed as we have every reason to expect it will be, from the eminence and competency of the source, cannot fail proving a valuable accession to our surgical literature.

*Prolapsus uteri.*—From an able paper, by Dr. J. T. Sharpless, of this city, on the *Use and abuse of the Pessary,* in the *Eclectic Journal* for Jan., we extract the following remarks on one of the protean causes of *procidentia uteri.*

"A fruitful secondary cause of uterine diseases, is, I fully believe, the system of tight dressing, with a stiff or even unyielding "bone" in front. The unnatural compression in *all* cases, of the internal organs by these "*straight jackets,*" is easily understood, whilst the support below being lessened by the increased cavity of the pelvis, and a diminution of the usual lateral protection by the width of the unribbed flank, render the lower viscera more likely to be forced down; and in this confined, or even compressed condition, they become, of course, more subject to disease. It is this *lateral* compressibility that gives our American ladies the reputation, over the world, for beauty of waist. Even the *front* bone is no doubt a source of mischief, for, in stooping, it presses into the abdomen just above the pubis, and its replacement, after apparent cure of Irritable Uterus, has, in several instances in my practice, produced serious relapses. For months, therefore, after *all* manifest disease has disappeared, I forbid the restoration of the "bone," or of lacing the jacket below the true ribs."

We may also add his judicious remarks on the pernicious effects of precocious marriages in the female. We have daily evidence of its baneful results.

"A frequent cause of chronic uterine disease is, too early maternity; and I think I am safe in saying, that a majority of marriages under seventeen, or indeed eighteen years of age in the female, is followed by some such derangement. The reason is easily understood. The immature structure is called to perform a duty, in a feeble condition that should only belong to a full development and perfection of health and strength, that, in our climate, does not appertain to that age."

---

DIED,

At Paris, on the morning of the 28th Oct., 1837, D. Franklin Hulme, M. D., of Philadelphia.

Suddenly, at Paris, on the 14th Nov., in the 21st year of his age, Jones Wistar, M. D., of Germantown, near Philadelphia.

Thus within a month of each other have two of our countrymen, pursuing their studies in the great metropolis of medicine, with every prospect of future success and reward, been suddenly cut off at the very onset of their career.

